# Research Progress on the Degradation of Organic Pollutants in Wastewater via Ultrasound/Periodate Systems: A Review

**DOI:** 10.3390/molecules29112562

**Published:** 2024-05-29

**Authors:** Tiehong Song, Zhe Wang, Yi Jiang, Shenggang Yang, Qiyuan Deng

**Affiliations:** Key Laboratory of Songliao Aquatic Environment, Ministry of Education, Jilin Jianzhu University, Changchun 130118, China; 17519465787@163.com (Z.W.);

**Keywords:** AOPs, activation mechanisms, cavitation effects, periodate, ultrasound

## Abstract

In recent years, the efficient removal of organic pollutants from wastewater has emerged as a critical area of global research interest. Against this backdrop, an array of innovative technologies for wastewater treatment has been developed. Among numerous advanced oxidation processes (AOPs), periodate (PI), an emerging oxidizing agent in AOPs, has garnered significant attention from researchers. Particularly, the integration of ultrasound (US)-activated PI systems has been recognized as an exceptionally promising approach for the synergistic degradation of organic pollutants in wastewater. In this paper, we conducted a thorough analysis of the mechanisms underlying the degradation of organic pollutants using the US/PI system. Furthermore, we comprehensively delineated the effects of ultrasonic power, periodate concentration, temperature, pH, coexisting inorganic ions, and dissolved organic matter on the removal efficiency of organic pollutants and summarized application cases of the US/PI system for the degradation of different pollutants. Finally, we also offered prospective discussions on the future trajectories of US/PI technology development.

## 1. Introduction

Concomitant with the relentless advance of modern industrialization, the diversity and concentration of organic pollutants discerned in wastewater have exhibited a discernible upward trajectory [1]. While traditional biological wastewater treatment technologies like adsorption, flocculation, and flotation have been pivotal in addressing a broad spectrum of wastewater challenges, their efficacy against certain recalcitrant organic pollutants remains limited [2,3]. This often leads to the release of inadequately treated effluents into natural waters and drinking water systems, posing significant environmental and health risks [4,5,6]. Consequently, the quest for wastewater treatment technology that is both efficient and comprehensive has assumed paramount importance.

Advanced oxidation processes (AOPs), epitomizing cutting-edge wastewater treatment paradigms, principally generate potent oxidizing free radicals in aqueous solutions via a constellation of chemical reactions [7,8]; this process facilitates the oxidation and decomposition of organic pollutants in water into inorganic substances, markedly mitigating or nullifying environmental harm [9]. As a critical component of advanced oxidation technologies, periodate possesses a substantial oxidation potential (1.6 V), endowing it with formidable oxidizing capabilities that render it particularly effective against recalcitrant pollutants. The oxidation reaction rate of periodate is not only moderate but also adjustable, qualities that significantly facilitate both experimental and industrial applications [10,11,12]. In comparison to more volatile oxidizing agents, periodate’s stable reactivity not only mitigates operational risks and minimizes by-product formation but also enhances overall safety. Toxicologically, periodates demonstrate reduced ecological and human toxicity compared to conventional oxidizers like chlorine and ozone [13], a characteristic that renders them particularly desirable in the context of contemporary environmental sustainability and safety priorities. Practically, the ready availability and cost-effectiveness of periodate on the market significantly enhance its utility [14]. Notably, periodate maintains consistent performance across a broad pH spectrum and is minimally influenced by pH variations, undoubtedly expanding its applicability and usage scenarios. Given these advantages, periodate-catalyzed oxidation technology has garnered substantial interest in scientific and industrial circles over recent decades, with its potential further amplified by escalating environmental standards and technological advancements [15,16,17]. Nonetheless, PI exhibits pronounced stability in aqueous solutions and resists facile activation for decomposition, constricting the genesis of free radicals with ample oxidizing prowess, thereby diminishing the efficacy of organic matter degradation in wastewater. Accordingly, devising strategies to efficaciously amplify the yield of reactive oxygen species (ROS) generated by PI has emerged as a pivotal challenge. Prior investigations have elucidated that the acoustic, optical, and electrochemical activation of PI can significantly elevate the ROS yield [18,19,20,21]. Nevertheless, photocatalytic methodologies have been eschewed owing to their suboptimal light utilization, inconsistent treatment efficiencies, and the potential biosafety concerns associated with ultraviolet radiation, whilst electrochemical approaches grapple with challenges related to diminished reactor efficiency and onerous operational protocols. Within this milieu, the ultrasound-activated PI approach is increasingly recognized as an innovative conduit to ameliorate the treatment of organic pollutants in wastewater, esteemed for its myriad merits, including high efficiency, environmental benignity, and operational convenience (Table 1).

The ultrasound-activated periodate (US/PI) technology has manifested remarkable proficiency in the degradation of organic pollutants, alongside notable environmental benefits within the domain of water treatment. Yet, detailed mechanistic elucidations and comprehensive, systematic reviews of this technology remain scarce. Consequently, there exists an imperative demand for an exhaustive treatise to bridge this void. This review delves into the following thematic areas: (a) an in-depth interpretation of the principles governing the degradation of organic pollutants in wastewater by the US/PI system; (b) a critical analysis of the primary factors influencing pollutant removal efficiency within the US/PI system; (c) a comprehensive evaluation of the degradation performance of the US/PI coupling technology across diverse organic compounds; and (d) an anticipatory exploration of the research development trends concerning the US/PI system. The objective of this review is to synthesize existing research findings and construct a robust theoretical framework that furnishes comprehensive theoretical underpinnings for future researchers to navigate and exploit this field efficaciously.

## 2. Reaction Mechanism of US/PI System for Degradation of Organic Pollutants in Wastewater

### 2.1. The Formation Mechanism of Oxidative Free Radicals in US/PI System

**Figure 1:**
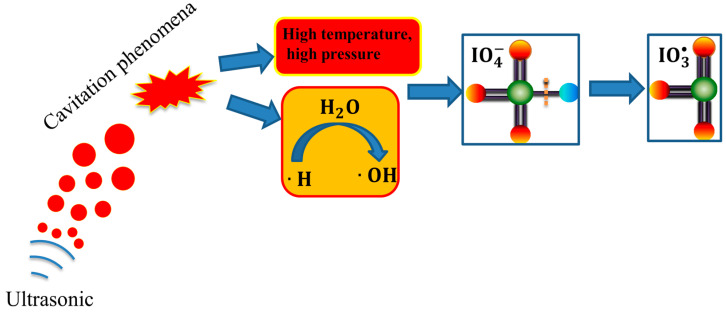
Schematic representation of the oxidation mechanism within the US/PI framework. (Herein, green symbolizes iodine atoms, orange symbolizes oxygen atoms, and blue denotes oxygen ions.).

The degradation efficiencies of pollutants within the US/PI system are predominantly attributed to a synergistic interplay between thermal decomposition and radical-driven oxidation processes [25]. In the initial stage, PI activation does not have a large amount of IO_4_^−^, resulting in less IO_3_^•^ free radicals. Consequently, pyrolysis occurring within the cavitation bubbles or in proximity to the gas–liquid interface emerges as the principal mechanism governing the decomposition of organic pollutants [22]. Furthermore, temperatures within the bubble or adjacent to the gas–liquid interface can escalate to approximately 5000 K during ultrasonic irradiation [26], and the significant elevation in temperature substantially augments the rate at which organic pollutants are degraded [27]. Concurrently, the collapse of cavitation bubbles within an aqueous solution generates exceptionally high temperatures and pressures, culminating in the thermal dissociation of water vapor into reactive hydroxyl radicals (•OH) and hydrogen radicals (•H) [28]. This process significantly enhances the oxidative decomposition of pollutants contained within the water. Subsequently, with the progression of time and the ultrasound-mediated activation of PI, there is a dramatic increase in the concentration of IO_3_^•^ radicals (Figure 1), which leads to a cascade of chemical reactions, culminating in the generation of a spectrum of reactive free radicals, which, in concert, constitute an acoustochemical degradation network (Equations (1)–(8)) [29]. Thus, the organic pollutants are effectively degraded, and finally, the organic matter is decomposed into CO_2_ and H_2_O, etc. Moreover, ultrasound induces significant turbulence within the aqueous solutions, thereby enhancing the mass transfer rates that are pivotal for the chemical reactions occurring in the solution [30]. It is widely acknowledged that IO_3_^•^ and •OH radicals represent the primary oxidizing agents within the US/PI framework [31]. Notably, the iodate ion (IO_3_^−^) invariably emerges as an intermediate product in the degradation of organic pollutants in wastewater treated with the US/PI system. The substantial presence of IO_3_^−^ mechanistically influences the degradation of organic matter; consequently, meticulous monitoring and precise quantification are imperative to ensure compliance with water quality safety standards and regulatory mandates. Presently, an array of established analytical techniques is employed for the identification and quantification of IO_3_^−^, ranging from traditional UV–visible and molecular absorption spectroscopy to contemporary ion chromatography and electrochemical detection methods, as well as titration techniques and the innovative laser-induced breakdown spectrometry (LIBS), all adept at accurately assessing the IO_3_^−^ concentrations in water samples [32]. While the World Health Organization has yet to establish a discrete limit for iodate in drinking water, it advocates that the aggregate iodine content, including iodate among other forms, should not surpass 150 µg/L to mitigate health risks associated with excessive iodine consumption. For the removal of IO_3_^−^, several efficacious methods are available, encompassing adsorption techniques, chemical reduction pathways, advanced oxidation processes, biodegradation mechanisms, and membrane separation techniques. In conclusion, the US/PI system effectively degrades organic pollutants, demonstrating substantial environmental benefits and extensive application potential [33].

(1) $$H_{2}O\overset{)))}{\to }•H+•\mathrm{OH}$$

(2) $$H•+{\;O}_{2}\overset{)))}{\to }HO_{2}^{•}$$

(3) $$HO_{2}^{•}\overset{)))}{\to }H^{+}+O_{2}^{•-}$$

(4) $${2H}_{2}O+\cdot H\overset{)))}{\to }HO_{2}^{•}+2H_{2}$$

(5) $$IO_{4}^{-}\overset{)))}{\to }IO_{3}^{•}+O^{•-}$$

(6) $$O^{•-}+H^{+}\overset{)))}{\to }•\mathrm{OH}$$

(7) $$•\mathrm{OH}+IO_{4}^{-}\overset{)))}{\to }IO_{4}^{•}+OH^{-}$$

(8) $${2H}^{+}+IO_{4}^{-}\overset{)))}{\to }IO_{3}^{•}+H_{2}O$$

### 2.2. Degradation Mechanisms of Different Organic Compounds in the US/PI System

Given the intricate and varied structures of organic pollutants in wastewater, attributing the degradation process in the US/PI system solely to the free radical mechanism oversimplifies the underlying dynamics; however, it is essential to consider the intermediates generated during the degradation process and their transformation pathways comprehensively, as these factors are pivotal in fully elucidating the degradation mechanism. Characterizing intermediates yields valuable insights for constructing logical reaction pathways and pinpointing the core mechanisms of action. Notably, the molecular structural characteristics and functional groups of the target organic compounds substantially shape the reaction dynamics within the US/PI system, underscoring the critical structure-reactivity relationship. Consequently, this paper summarizes key findings on the degradation mechanisms of diverse organic pollutants in the US/PI system (Table 2).

In summary, US/PI technology demonstrates highly intricate and varied degradation mechanisms across different organic compounds, underscoring the imperative for further studies to enrich the theoretical base and detail the mechanisms involved. To advance the frontier of knowledge in this domain, the authors advocate for a deeper investigation of these intricate reaction mechanisms through a blend of experimental validation and theoretical calculations. Through such multidimensional explorations, we anticipate unlocking the potential of US/PI technology, optimizing its application in wastewater treatment, and providing a robust scientific foundation for addressing the challenges posed by recalcitrant organic pollutants in the environment.

## 3. Key Determinants of the US/PI System Is Performance

While the US/PI system presents a promising avenue for the efficacious remediation of organic contaminants in wastewater, its performance is contingent upon myriad factors, such as ultrasonic intensity, periodate dosage, ambient temperature, solution pH, the presence of inorganic co-ions, and dissolved organic matter. To furnish readers with a deeper understanding of the underlying mechanisms governing these factors, a comprehensive discussion is delineated in the ensuing sections.

### 3.1. Ultrasonic Power Intensity

**Figure 2:**
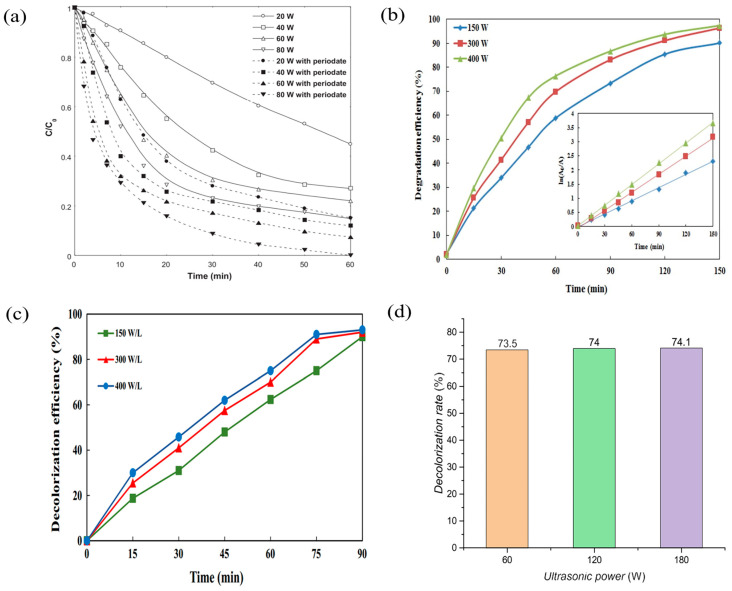
The influence of ultrasonic power on the degradation efficiency across various dye types. (**a**) The impact of varying levels of ultrasonic power on the degradation efficiency of Bright Blue R dye [40]. Reproduced with permission from Elsevier. (**b**) The progression of degradation for Acid Blue 92 dye under disparate ultrasonic power [35]. Reproduced with permission from Elsevier. (**c**) The governing principles underlying the influence of ultrasonic power on the decomposition process of Acid Orange 7 dye [37]. Reproduced with permission from Elsevier. (**d**) The influence of ultrasonic power on the degradation efficiency of Methylene Blue Dye [43]. Reproduced with permission from Elsevier.

The intensity of power significantly influences the efficacy of the US/PI system in degrading organic pollutants in wastewater [39]. Hamdaoui et al. [40] varied the power levels (20 W, 40 W, 60 W, 80 W) to conduct degradation experiments on Brilliant Blue R. They set the initial dye concentration at 5 mg/L, the PI concentration at 10 mM, the temperature at 25 °C, and the pH at 5.2. Within a span of 60 min, there was a significant enhancement in the removal efficiency of organic matter, which correlated positively with an increase in power (Figure 2a). Similarly, Khataee et al. [35] conducted decolorization experiments on Acid Blue 92 dye, utilizing three distinct ultrasonic power settings: 150 w, 300 w, and 400 w. The initial dye concentration was set at 10 mg/L, while the concentration of PI was established at 1 g/L. Over the course of 90 min, the degradation efficiencies achieved were 73.35%, 84.14%, and 87.71%, respectively (Figure 2b). The rationale behind these findings is that an escalation in the power intensity of ultrasound engenders a surge in the production of reactive free radicals, thereby amplifying the removal of organic pollutants [41]. Furthermore, an increase in ultrasonic power intensifies the turbulence within the solution, which in turn enhances the mass transfer rate and consequently, the degradation efficiency [42].

However, the escalation of ultrasonic power is not without its limitations concerning pollutant removal efficacy. Khataee et al. [37] conducted decolorization experiments on Acid Orange 7 dye, employing ultrasonic powers of 150 W/L, 300 W/L, and 400 W/L. It was determined that the ultrasonic power setting of 150 W/L was the most efficacious for the experiment. The initial dye concentration was established at 5 mg/L, with the PI concentration set at 1 g/L, and the pH maintained at 6.34. Over a period of 60 min, an increase in power correlated with enhanced decolorization efficiency. However, extending the reaction time to 90 min resulted in negligible differences in decolorization efficiency among the three power settings (Figure 2c). Sukhatskiy et al. [43] conducted a series of degradation experiments on methylene blue across varying power conditions: C_0_ = 62.6 μMol, pH = 7.00, and an initial temperature of 17.0 °C. The study revealed that increasing the power from 60 W to 180 W exerted a negligible influence on the decolorization efficiency of the MB aqueous solution, manifesting a mere enhancement of 0.6% (from 73.5% to 74.1%) (Figure 2d). This phenomenon can be attributed to the initial stages, where an incremental increase in ultrasound energy intensity facilitates the effective generation of more active free radicals, leading to a significant enhancement in pollutant degradation. However, upon reaching a certain ultrasound power threshold, the excess bubbles produced through cavitation cease to contribute effectively to the degradation process and instead escape from the solution, dispersing to the vessel wall or reversing the transmission through the ultrasound transducer, thereby intensifying the ineffective loss of ultrasound energy. Nevertheless, the actual effective energy output remains unchanged, leading to the observed insignificance in degradation efficacy among different power settings at this stage [44].

In summary, while enhanced ultrasonic power can indeed accelerate the degradation of specific organic pollutants, its effectiveness is not indefinite; rather, it tends to plateau or even diminish beyond a certain saturation point. The full utilization of ultrasonic technology hinges on a comprehensive understanding of its operational mechanisms, particularly in terms of precisely regulating the power levels to optimize purification efficiency. This study explores the modulation of chemical and physical reaction kinetics with ultrasonic power and investigates its intricate effects on the dynamic behaviors of cavitation phenomena. Practically, the substantial energy consumption and associated costs of high-power ultrasound treatments present significant constraints. Consequently, it is essential to precisely calibrate the power settings to strike an optimal balance between treatment efficacy and economic feasibility. Future studies should aim to identify the optimal ultrasound power settings for varying types of pollutants and chemical contexts, to delineate the most effective point on the power–efficiency curve. Moreover, comprehensive investigations into the long-term performance stability, efficiency progression, and equipment durability of ultrasonic treatment are indispensable. Thorough cost–benefit analyses, encompassing energy consumption and operational and maintenance expenses, are crucial to evaluate the practical viability and sustainability of ultrasound technology in large-scale industrial settings.

### 3.2. PI concentration Levels

**Figure 3:**
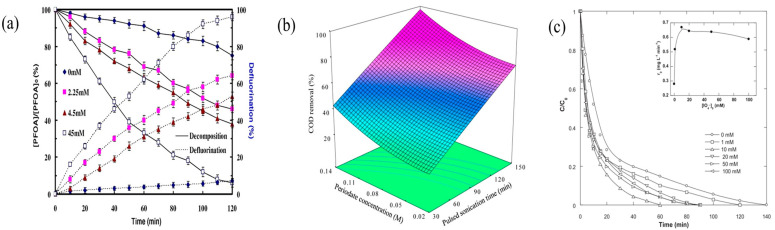
PI concentration on the degradation efficiency across a spectrum of targeted pollutants. (**a**) The trajectory of PFOA degradation efficiency as a function of escalating PI concentration [34]. Reproduced with permission from Elsevier. (**b**) The efficacy of pollutant removal in authentic industrial wastewater subjected to varying PI concentrations [45]. Reproduced with permission from Elsevier. (**c**) The associated modifications in the degradation rate of the dye Brilliant Blue R consequent to differing PI concentrations [40]. Reproduced with permission from Elsevier.

The concentration of PI plays a pivotal role in enhancing the removal efficiency of pollutants within the US/PI system. Lee et al. [34] conducted degradation experiments on perfluorooctanoic acid using various concentrations of PI (0, 2.25, 4.5, and 45 mmol/L). The initial concentration of PFOA was set at 170.1 μM, with the pH starting at 3.9. The findings indicate that there was a corresponding increase in degradation efficiency concomitant with the elevation in PI concentration (Figure 3a). Similarly, Moradian et al. [36] executed the degradation of real waste permeate under diverse PI concentration regimes with the experimental findings validating a pronounced diminution in BOD5 and COD levels concurrent with the escalation of oxidant concentration. Moreover, Darvishi Cheshmeh Soltani et al. [45] utilized varying concentrations of PI (0.02, 0.05, 0.08, 0.11, and 0.14 mmol/L) to treat industrial wastewater. The duration of pulsed ultrasound ranged from 30 to 150 min, with the process temperature maintained at 313 K. The experimental evidence unequivocally demonstrates that an increase in PI concentration not only facilitates the improvement in COD removal but also contributes to the reduction in the required ultrasonication time (Figure 3b). The observed phenomenon can be attributed to the fact that an elevated concentration of PI in the solution precipitates an augmentation in IO_4_^−^ levels. This increment, in turn, fosters the generation of an increased number of IO_3_^•^ radicals, thereby significantly enhancing the degradation efficiency of organic pollutants.

Moreover, an excessively high concentration of PI can paradoxically impair the removal efficiency of certain organic pollutants. For instance, Hamdaoui et al. [40] investigated the degradation of Brilliant Blue R across a PI concentration range of 1 to 100 mmol/L. The initial dye concentration was 5 mg/L, the initial temperature was 25 °C, and the initial pH was 5.2. Investigations revealed that a PI concentration of 10 mmol/L facilitated the completion of the degradation within 60 min. Conversely, a PI concentration of 100 mmol/L necessitated a duration of 90 min to achieve complete degradation (Figure 3c). Similarly, Seid-Mohammadi et al. [46] conducted phenol removal studies with PI concentrations ranging from 1 mmol/L to 7 mmol/L. Investigations revealed that elevating the periodate concentration from 1 mmol/L to 2 mmol/L resulted in an enhancement in PI removal efficiency from 86.2% to 93.6%. Nonetheless, a subsequent increase in periodate concentration was observed to inversely affect the removal efficiency. This phenomenon can be attributed to excess IO_4_^−^, leading to a free radical surge that depletes strongly oxidizing radicals (IO_3_^•^, •OH) (Equations (9) and (10)).

(9) $$•\mathrm{OH}+IO_{4}^{-}\overset{)))}{\to }IO_{4}^{•}+OH^{-}$$

(10) $$IO_{3}^{•}+IO_{4}^{-}\overset{)))}{\to }IO_{3}^{-}+IO_{4}^{•}$$

An additional factor involves the reactivity among free radicals themselves, resulting in a diminished yield of reactive radicals and, consequently, a reduction in the organic pollutant’s degradation efficiency (Equations (11)–(13)) [46].

(11) $$2•\mathrm{OH}\overset{)))}{\to }H_{2}O_{2}$$

(12) $$2IO_{3}^{•}\overset{)))}{\to }I_{2}O_{6}$$

(13) $$2IO_{4}^{•}\overset{)))}{\to }I_{2}O_{8}$$

In summary, the adjustment of the PI solution concentration plays a pivotal role in the degradation process of pollutants. Optimal concentration levels significantly enhance the decomposition rates of pollutants, whereas suboptimal concentrations may attenuate the treatment efficacy or even exert adverse effects. This observation underscores the critical imperative for precise control over chemical concentrations in environmental treatment and wastewater purification. Consequently, there is a pressing necessity to develop and refine sophisticated modeling systems that not only predict but also precisely guide the optimal PI concentration settings to maximize pollutant removal efficacy, thereby advancing environmental treatment technologies towards greater efficacy and sustainability.

### 3.3. Temperature

**Figure 4:**
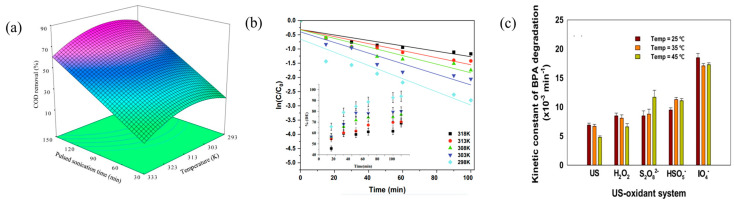
Temperature variations on the degradation efficiency of selected pollutants across various types of industrial wastewater. (**a**) Variations in the overall degradation performance of industrial wastewater subjected to differing temperatures [45]. Reproduced with permission from Elsevier. (**b**) The efficacy of Ramazol Black B removal under varying temperature [48]. Reproduced with permission from Elsevier. (**c**) The influence of temperature fluctuations on the degradation efficiency of Bisphenol A within wastewater treatment systems [24]. Reproduced with permission from Elsevier.

In the US/PI system toward organic pollutants treatment, reaction temperature emerges as a crucial parameter influencing solution viscosity and the mass transfer coefficient. Safari et al. [45] conducted degradation experiments on industrial wastewater at varied temperatures. The findings demonstrate that an elevation in temperature from 293 K to 313 K results in a substantial augmentation in the efficiency of COD removal (Figure 4a). Similarly, Sponza et al. [38] explored phenol degradation at various temperatures, discovering a significant uptick in the removal of phenol derivatives as the temperature escalated. This phenomenon can be attributed to the enhanced molecular motion of the oxidant as temperature increases, which subsequently elevates the concentration of active free radicals, thereby accelerating the reaction rate [47].

Previous studies have also identified a negative correlation between the removal of organic pollutants and higher reaction temperatures. For instance, Ahmadi et al. [48] conducted a series of kinetic degradation experiments on Rabeprazole Black B using the US/PI system at various temperatures (298 K, 303 K, 308 K, 313 K, and 318 K). The initial concentration was set at 100 mg/L, with PI concentration at 0.35 g/L, and pH maintained at 3. The outcomes revealed a decrement in the reaction rate constant concurrent with an escalation in temperature (Figure 4b). Furthermore, Sun et al. [24] conducted degradation experiments on Bisphenol A at varying temperatures (25 °C, 35 °C, and 45 °C), employing a frequency of 35 kHz and power of 58 W. The initial concentration of Bisphenol A was 0.0376 mmol/L, and the PI concentration was 0.338 mmol/L. The findings indicated that there was a reduction in degradation efficiency as the temperature increased (Figure 4c). The observed phenomenon can be attributed to the enhanced degassing of the solution under specific high-temperature conditions, which diminishes the availability of gas nuclei for forming cavitation bubbles [49], thereby decreasing the yield of active free radicals. At lower temperatures, the solution exhibits decreased vapor pressure and increased viscosity, which facilitate the cavitation generation and enhance the yield of free radicals. Consequently, low temperatures may enhance the degradation of organic pollutants, whereas high temperatures may impede their removal.

In conclusion, a moderate elevation in temperature generally promotes the degradation rate of many organic pollutants; however, an excessive rise can inversely impact this process. It is crucial to acknowledge that these findings, derived from a subset of organic pollutants, require broader generalization and robust validation. Future studies should aim to broaden the scope to include a diverse array of compounds and wastewater samples, thereby enhancing our comprehension of the temperature effect mechanisms. Concurrently, the meticulous documentation of experimental parameters and conditions is essential to guarantee the reproducibility of findings and their comparability across different studies. Additionally, advancing the development of sophisticated predictive models to accurately estimate and customize the optimal response temperature for various pollutant properties and treatment scenarios is pivotal in enhancing the efficacy and precision of environmental management strategies.

### 3.4. Solution pH Value

Within the US/PI co-treatment system, pH assumes a pivotal role, markedly influencing both the efficiency and kinetics of the organic pollutant degradation process. Across a variety of organic pollutants, optimal pH conditions exhibit substantial variability (Table 3). To elucidate the mechanism underlying the pH-induced degradation of organic pollutants more comprehensively, the authors provide an in-depth exploration and illustrative examples herein.

**Figure 5:**
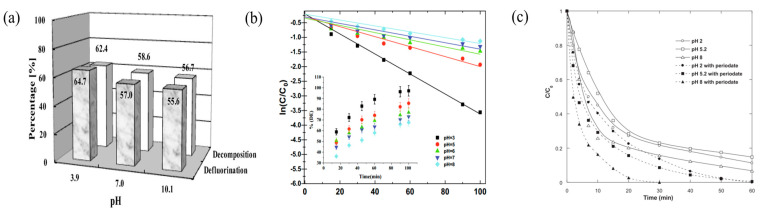
pH variability on the degradation efficiency across pollutants. (**a**) Varied degradation rates of perfluorooctanoic acid (PFOA) across pH spectra [34]. Reproduced with permission from Elsevier. (**b**) The degradation trajectory of Ramazol Black B under diverse pH scenarios [48]. Reproduced with permission from Elsevier. (**c**) The pronounced impact of pH on the removal efficiency for the dye Brilliant Blue R [40]. Reproduced with permission from Elsevier.

Increasing solution acidity is shown to enhance the removal of many organic pollutants [50]. Lee et al. [34] studied PFOA degradation across pH levels (3.9, 7.0, 10.1). When the concentration of PFOA = 170.1 μMol, PI = 4.5 mmol/L. The analysis revealed a discernible downtrend in degradation efficiency as pH levels ascended, demonstrating that the peak decomposition rate of 62.4% was attained at pH 3.9, subsequently diminishing to the nadir of 56.7% at pH 10.1 (Figure 5a). This phenomenon is due to increased positive charge density in the gas–liquid interface below pH 7, thereby reducing bubble aggregation and attracting oppositely charged free radicals, enhancing PFOA’s acoustic chemical degradation. Acidic conditions, through acid catalysis, boost the generation of IO_3_^•^ radicals, thus improving degradation efficiency. Seid-Mohammadi et al. [46] carried out a series of phenol degradation experiments across varying pH levels (3, 7, and 11). The experimental outcomes demonstrated a progressive decline in the phenol degradation rate with an increase in solution pH. Lower pH levels lead to higher hydrogen ion concentrations, spurring the creation of •OH and IO_3_^•^ radicals, significantly boosting phenol degradation efficiency. Ahmadi et al. [48] conducted degradation treatments of Ramazol Black B at different pH values, where C_0_ = 100 mg/L, catalyst dosage = 0.15 g/L, and T = 298 K, and the results indicated that the degradation efficiency was optimal at pH = 3 (Figure 5b). Moradian et al. [36] observed similar outcomes in the degradation of actual waste permeate. This reaffirms that acidic environments favor the decomposition of most organic pollutants in the US/PI system.

Notwithstanding, our findings also illuminate that for a subset of organic pollutants, degradation efficacy progressively augmented as the environmental pH transitioned from acidic to alkaline. In their seminal study, Hamdaoui et al. [40] probed the degradation dynamics of Brilliant Blue R across varying pH levels (2, 5.2, and 8). The initial dye concentration was 5 mg/L, the initial PI concentration was 10 mMol, the initial temperature was 25 °C, the initial frequency was 300 kHz, and the initial ultrasonic power was 80 W. The findings demonstrated that under alkaline conditions at a pH of 8, the degradation efficiency reached up to 97% within 60 min, significantly surpassing the efficiencies recorded at 77% and 84% for pH levels of 2 and 5.2 (Figure 5c). Analogously, Sun et al. [24] embarked on a series of degradation assays involving Bisphenol A at diverse pH settings, discerning a concomitant elevation in degradation efficiency parallel to the ascension in pH. The genesis of this phenomenon can be primarily ascribed to alterations in the scavenging rates of IO_3_^•^ radicals by IO_4_^−^ across disparate pH landscapes. Specifically, the accelerated reactivity between IO_3_^•^ and IO_4_^−^ in acidic milieux precipitates a heightened relative concentration of IO_3_^•^ in alkaline settings, culminating in an elevated degradation efficiency for organic pollutants owing to IO_3_^•^’s pivotal role as the principal oxidizing agent in the decomposition of organic substrates. Furthermore, the activation of periodate via •OH radicals under mildly alkaline conditions [51] engenders the formation of singlet oxygen, a potent oxidizer, thereby amplifying the degradation prowess of organic entities. Consequently, for certain distinct organic pollutants, an environment with moderate alkalinity emerges as more conducive to their degradation pathway.

In conclusion, the role of pH is pivotal in determining the efficiency of the US/PI system for purifying organic pollutants from wastewater. This is particularly evident in the comparative analysis of acidic and alkaline conditions, which underscores the specific and significant impact of environmental pH on treatment efficacy. To enhance the applicability and practical significance of these findings, future studies should broaden their scope to investigate how various organic pollutants respond differently to pH changes. Additionally, it is crucial to ensure the consistency of experimental designs and to assess the stability of long-term operations more rigorously. Furthermore, a deeper investigation into the degradation mechanisms of various organic pollutants across different pH environments is essential. Identifying the key factors that influence the efficiency of these degradation pathways is crucial for enhancing the effectiveness of treatment technologies. Building on this foundation, developing more precise pH control strategies and techniques will allow for the tailored adjustment of pH levels to meet the specific needs of pollutants in practical sewage treatment scenarios, thereby maximizing pollutant removal efficiency and advancing water treatment technologies to a new level of higher efficiency and greater intelligence.

### 3.5. Presence of Concurrent Inorganic Ions

Inorganic ions assume a pivotal and multifaceted role in catalyzing the degradation reactions of diverse organic pollutants. Notably, ions such as Cl^−^, HCO_3_^−^, CO_3_^2−^, NO_3_^−^, SO_4_^2−^, and H_2_PO_4_^−^ are universally acknowledged for their characteristic inhibitory impacts; yet, it is crucial to underscore that not every inorganic ion hampers the degradation of organic pollutants. In contrast, a specific investigation illuminated that Br^−^ [34], representing a noteworthy exception, significantly augments the degradation efficiency of organic pollutants. This phenomenon will be further expounded and meticulously analyzed in the subsequent section.

In the analyzed anion systems, Cl^−^ forms Cl^•^ radicals via oxidation, yet its oxidation of organic compounds is markedly less efficient than CO_3_^•−^ [52]. Consequently, Cl^−^ more effectively inhibits organic matter degradation than both HCO_3_^−^ and CO_3_^2−^. Previous studies show that CO_3_^2−^ reacts with •OH 46 times faster than HCO_3_^−^ [53], indicating that CO_3_^2-^ has greater efficacy in hindering degradation compared to HCO_3_^−^. Conversely, SO_4_^2−^ is less inhibitory than HCO_3_^−^ and CO_3_^2−^ due to the higher oxidizing ability of SO_4_^•−^ and S_2_O_8_^2−^ generated during hydroxyl radical reactions, which can enhance the degradation of some organic dyes [54]. NO_3_^−^ and H_2_PO_4_^−^ show minimal organic matter degradation in the US/PI system, primarily because NO_3_^−^ ’s slow reaction with •OH radicals leads to an insignificant reduction in reactive radicals, rendering its inhibitory effect negligible [55]. The inhibitory effect of H_2_PO_4_^−^ on organic degradation is notably weak, as its reaction with •OH radicals yields the less reactive H_2_PO_4_^•^ radical at a slow rate [56]. In conclusion, the sequence of inorganic anions inhibiting organic matter degradation is approximately Cl^−^ > CO_3_^2−^ > HCO_3_^−^ > SO_4_^2−^, with NO_3_^−^ and H_2_PO_4_^−^ exerting minimal effects. The chemical reactions underpinning these findings are detailed in (Equations (14)–(23)).

(14) $$•\mathrm{OH}+Cl^{-}\overset{)))}{\to }OH^{-}+Cl^{•}$$

(15) $$Cl^{-}+Cl^{•}\overset{)))}{\to }{\mathrm{CI}}_{2}^{•-}$$

(16) $$•\mathrm{OH}+Cl^{-}+H^{+}\overset{)))}{\to }H_{2}O+Cl^{•}$$

(17) $$\mathrm{HC}O_{3}^{-}+•\mathrm{OH}\overset{)))}{\to }CO_{3}^{•-}+H_{2}O$$

(18) $$CO_{3}^{2-}+•\mathrm{OH}\overset{)))}{\to }CO_{3}^{•-}+OH^{-}$$

(19) $$SO_{4}^{2-}+•\mathrm{OH}\overset{)))}{\to }SO_{4}^{•-}+OH^{-}$$

(20) $$SO_{4}^{•-}+H_{2}O\overset{)))}{\to }SO_{4}^{2-}+•\mathrm{OH}+H^{+}$$

(21) $$SO_{4}^{•-}+SO_{4}^{•-}\overset{)))}{\to }S_{2}O_{8}^{2-}$$

(22) $$•\mathrm{OH}+NO_{3}^{-}\overset{)))}{\to }NO_{3}^{•}+OH^{-}$$

(23) $$•\mathrm{OH}+H_{2}PO_{4}^{-}\overset{)))}{\to }H_{2}PO_{4}^{•}+OH^{-}$$

**Figure 6:**
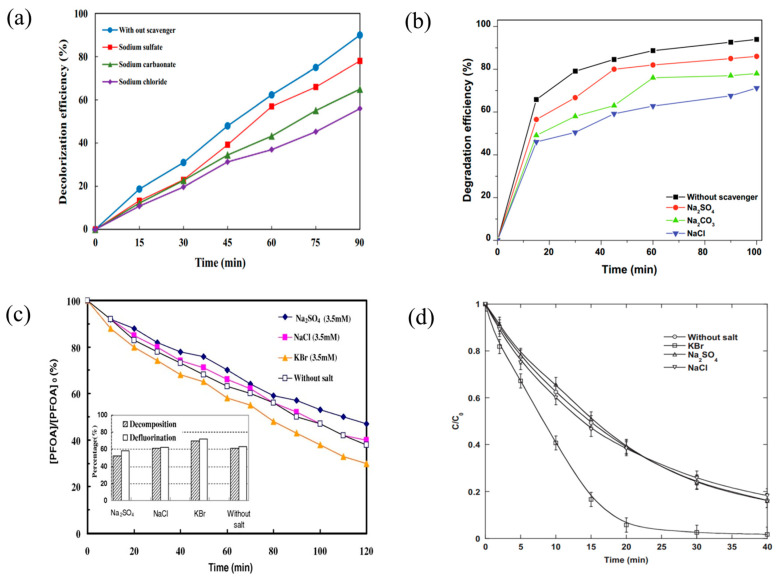
The Impact of Varied Ions on Pollutant Degradation Efficiency: (**a**) the comparative effects of Na_2_SO_4_, Na_2_CO_3_, and NaCl on Acid Orange 7 degradation [37]. Reproduced with permission from Elsevier. (**b**) Analysis of sodium sulfate, sodium carbonate, and sodium chloride on Ramazol Black B dye removal [48]. Reproduced with permission from Elsevier. (**c**) The contribution of KBr to the decomposition of PFOA and its influence on fluoride removal efficiency [34]. Reproduced with permission from Elsevier. (**d**) The role of bromide ions, as represented by KBr, in enhancing the degradation of Malachite Green [57]. Reproduced with permission from Elsevier.

Thangave and colleagues [52] explored the impact of various anions (Cl^−^, HCO_3_^−^, H_2_PO_4_^−^, and SO_4_^2−^) on the degradation efficiency of Reactive Red 120 (RR120) dye. They observed a variable reduction in efficiency upon introducing these anions, with the order of inhibitory effect being Cl^−^ > HCO_3_^−^ > SO_4_^2−^ > H_2_PO_4_^−^. Khataee et al. [37] reported that in experiments with Acid Orange 7 (AO7) under specific conditions (dye concentration of 5 mg/L and reaction time of 90 min), the addition of Na_2_SO_4_, Na_2_CO_3_, and NaCl decreased the decolorization rate of AO7 from 90% to 78%, 65%, and 56%, respectively (Figure 6a). Likewise, Abbas Rahdar and his team [48] noted that adding Na_2_SO_4_, Na_2_CO_3_, and NaCl to Ramazol Black B solutions inhibited its degradation, with the degree of inhibition ranking as Cl^−^ > CO_3_^2−^ > SO_4_^2−^ (Figure 6b). The catalyst dosage = 0.35 g/L, C_0_ = 100 mg/L, scavenger concentration = 10 mg/L, pH = 3, and T = 298 K. In conclusion, these experiments collectively offer robust evidence supporting prior findings and confirm the specific anions’ inhibitory effects on the degradation of organic pollutants.

However, the influence of inorganic anions on the degradation of organic pollutants is not universally inhibitory. Lee et al. [34] uncovered a contrasting phenomenon in their study on PFOA treatment. In their degradation of perfluorooctanoic acid, they found that the addition of Br^−^ ions actually significantly enhanced the decomposition rate and fluoride removal efficiency of the substance, and the degradation efficiency increased with increasing Br^−^ concentration (Figure 6c). Similarly, Moumeni [57] et al. showed that bromide ions had a catalytic accelerating effect during the chemical degradation of malachite green using ultrasonic techniques (Figure 6d).

The promotive influence of Br^−^ ions originates from their capacity to infiltrate the cavitation vesicle’s interfacial region at elevated concentrations, thereby facilitating and catalyzing free radical generation. More specifically, Br^−^ engages with the hydroxyl radical (•OH) in a sequence of reactions to form the radical anion Br_2_^•−^ (Equations (24)–(27)), thereby amplifying the organic matter degradation reaction.

(24) $$Br^{-}+•\mathrm{OH}\overset{)))}{\to }\mathrm{BrO}H^{•-}$$

(25) $$\mathrm{BrO}H^{•-}\overset{)))}{\to }Br^{•}+OH^{-}$$

(26) $$\mathrm{BrO}H^{•-}+Br^{-}\overset{)))}{\to }Br_{2}^{•-}+OH^{-}$$

(27) $$Br^{•}+Br^{-}\overset{)))}{\to }Br_{2}^{•-}$$

The dibromo radical anion Br_2_^•−^ stands out as a potent oxidizing entity, characterized by a noteworthy redox potential of 1.63 V and maintaining high stability at concentrations of up to 105 mol/L in solution. This attribute enables Br_2_^•−^ to efficaciously engage with organic compounds at the cavitation vesicle surface, consequently augmenting the degradation efficacy of the organic material. Moreover, the action mechanism of Br^−^ ions in degrading organic matter adheres to the Hofmeister effect, delineating a specific affinity towards the gas–liquid interface. Relative to the bulk of the solution, Br^−^ ions exhibit a propensity to aggregate at the bubble interface, creating a pronounced negative charge distribution. This phenomenon not only boosts the ion concentration at the gas–liquid interface but also amplifies the electrostatic repulsion among cavitation bubbles, leading to their division into smaller entities and concurrently expanding the surface area of organics subjected to pyrolysis. Consequently, the presence of Br^−^ ions facilitates the efficient decomposition of select organic pollutants through the amplification of the cavitation effect and the enlargement of the pyrolytic contact area.

It is evident that inorganic anions significantly influence the degradation of organic pollutants in the US/PI system, with the action of bromide ions (Br^−^) being particularly pronounced under certain environmental conditions. To enhance the depth of understanding and expand its applicability, this study should extend its analysis to a broader spectrum of inorganic anions and explore a wider array of environmental parameters, thereby augmenting the generalizability and practical utility of the findings. Concurrently, the formulation of any treatment strategies must integrate considerations of environmental sustainability and economic feasibility to ensure that the technology not only safeguards ecological integrity but also remains economically viable, thereby achieving the sustainable management of environmental pollution.

### 3.6. Dissolved Organic Matter

Beyond inorganic ions, the influence of dissolved organic matter on the efficacy of the US/PI system in wastewater treatment is significant and cannot be overlooked. When Thangavel et al. [52] introduced tert-butanol into the RR120 dye solution, there was a notable decrease in the degradation efficiency of the dye. Seid-Mohammadi et al. [46] incorporated TBA to attenuate the removal effect during the phenol degradation process. The results indicated that TBA addition substantially diminished phenol decomposition. The observed phenomenon can be attributed to the role of tert-butanol as a scavenger of the •OH free radical, leading to a reduction in the number of active free radicals within the system and consequently a significant drop in removal efficiency. Furthermore, humic acid (HA) plays an indispensable role in the efficacy of ultrasound-activated PI oxidation. Reports indicate that with 17.5 to 35 mg/L of humic acid, bisphenol A degradation is significantly hindered due to substantial competition for active free radicals. [58].

However, the capability of dissolved organic matter to inhibit pollutant degradation often presents limitations. Sponza et al. [38] examined the impact of adding perfluorohexane (C_6_F_14_) on the removal efficiency during the degradation of industrial organic wastewater. The results demonstrated that at a concentration of 19 mg/L of C_6_F_14_, the removal rate for TAAs increased to 90%, and for phenol to 98%. This effect can be attributed to the low boiling point and low water solubility of C_6_F_14_, which causes it predominantly to exist in the gas phase. Additionally, as these highly halogenated compounds react with hydrogen atoms to remove phenol, this reaction leads to an increased concentration of OH radicals. Consequently, this results in enhanced degradation efficiency of organic matter in wastewater.

The role of dissolved organic matter (DOM) in the ultrasonic/persulfate (US/PI) system manifests as a complex, dualistic force, simultaneously impeding and facilitating pollutant degradation. This paradox underscores the profound complexities inherent in devising and implementing wastewater treatment strategies, necessitating a nuanced and thorough comprehension of the system. Presently, the understanding of dissolved organic matter behavior within this system remains limited. Consequently, future research should aim to expand the scope and deeply investigate the impact of various dissolved organic matter species on the US/PI system’s mechanisms, aiming to elucidate the potential beneficial or detrimental patterns. Simultaneously, developing innovative strategies to proactively regulate or adeptly exploit the unique properties of dissolved organic matter, thereby leveraging these characteristics to enhance wastewater treatment efficacy, represents a crucial avenue for technological progress in this field. Through these scientific endeavors, we aim to discover novel and efficient methods for wastewater purification to address the escalating environmental challenges.

### 3.7. Others

A multitude of additional factors significantly influence the organic degradation process in the treatment of organic pollutants in wastewater using the US/PI ion system [59], encompassing, among others, the type of liquid medium and the concentration of dissolved oxygen. More specifically, the facilitation of acoustochemical reactions exhibits notable variations across different types of ultrasonic fluids. Due to their low surface tension and elevated vapor pressure, organic solvents are generally considered to impede the efficient progression of sonochemical reactions. Consequently, considering these factors, aqueous solutions are preferentially selected as the reaction medium for sonication in practical applications [43]. Additionally, the concentration of dissolved oxygen within aqueous solutions emerges as a critical factor that warrants careful consideration. Under specific conditions, a reduced concentration of dissolved oxygen is actually conducive to amplifying the efficacy of acoustochemical reactions, namely, enhancing acoustic dissolution efficiency. This phenomenon can be attributed to the activation of specific redox reaction pathways, which are more readily facilitated in low-oxygen environments, thereby augmenting the degradation process of organic matter [60].

In summary, the nature of the solvent and the concentration of dissolved oxygen critically influence the efficacy of sonochemical reactions. Specifically, aqueous solutions demonstrate enhanced performance compared to other solvents in sonochemical reactions. It is widely accepted that reducing dissolved oxygen content may improve the efficiency of oxidation reactions; however, the optimization of this effect requires further investigation. Future research should concentrate on elucidating the specific mechanisms by which changes in dissolved oxygen concentration impact sonochemical reactions and on identifying optimal conditions for reducing dissolved oxygen levels. Moreover, examining the detailed influences of solvents with diverse chemical properties, including polar, non-polar, and ionic solvents, on sonochemical reactions will underpin the rational selection of solvents, thereby optimizing and broadening the utility of these reactions in practical applications. Through meticulously designed experiments, we can acquire a profound understanding of how these variables synergistically enhance reaction efficiency.

## 4. Efficacy of Organic Compound Degradation under the US/PI System

As delineated in preceding research [61,62,63], ultrasound technology has been validated to proficiently activate an array of potent oxidizing agents—including hydrogen peroxide, periodate, and Fenton’s reagents—thereby augmenting the eradication efficiency of organic pollutants in wastewater. Nonetheless, the efficacy of free radical generation via hydrogen peroxide under ultrasonic conditions is comparatively modest [64], and ultrasonically excited Fenton’s reagents are susceptible to undergoing complexation reactions within the solution, consequently impeding their performance [65]. In contrast, the remarkable radical generation capability and energy efficiency of ultrasound-activated periodate have garnered significant attention from the scientific community [11]. Presently, the integrated US/PI system is extensively employed in the degradation of a diverse array of pollutants, including but not limited to dyes [40], industrial wastewater [45], pharmaceutical compounds [66], endocrine disruptors [24], perfluorinated compounds [34], etc. (Table 4).

Clearly, for identical pollutants, the degradation efficiency of the US/PI system is significantly enhanced relative to that of the standalone systems. Additionally, the reaction time required is considerably shorter. For instance, under the US system alone, Brilliant Blue R achieves 100% degradation in 140 min, whereas under the US/PI system, it is completely degraded in just 60 min. Similarly, Acid Orange 7 shows a degradation rate of 27.5% in 105 min under the US system alone, compared to 96% in just 90 min under the US/PI system. Evidently, the US/PI coupling system demonstrates a more profound degradation effect compared to either ultrasonic or periodate degradation alone, achieving a degradation efficiency exceeding 94% and a mineralization efficiency of over 77%. This pronounced disparity stems from the reliance of the standalone ultrasonic degradation process on transient high temperatures induced through ultrasound’s thermal effects and the oxidation of organic substances via •OH radicals [67]. However, the efficacy of ultrasonic degradation in isolation is suboptimal, attributable to the constrained production of •OH radicals. Conversely, PI exhibits high stability in pure aqueous solutions, is resistant to spontaneous decomposition, and seldom engenders potent oxidizing free radicals such as IO_3_^•^ [68]. Consequently, its solo degradation efficiency for organic pollutants is markedly low. Within the synergistic US/PI framework, ultrasonic cavitation triggers the rapid establishment of a high-temperature, high-pressure milieu within the solution, markedly expediting the conversion of IO_4_^−^ into IO_3_^•^. Concurrently, the intense turbulent motion engendered by ultrasound propagates through the solution, substantially elevating the mass transfer rate of chemicals therein [30]. Furthermore, the oxidation reactions within this system act as a catalyst for generating a spectrum of active free radicals—including IO_3_^•^, IO_4_^•^, and •OH—working in concert to substantially enhance the degradation efficacy of organic pollutants in wastewater [57,69].

In summary, US/PI technology has demonstrated outstanding efficacy in treating organic pollutants in water, significantly reducing the presence of harmful compounds. The substantial benefits it offers in enhancing water quality and safeguarding both aquatic ecosystems and human health are undeniable. Although this technology achieves high degradation efficiency, the complete mineralization of certain organic compounds remains suboptimal, presenting a significant limitation in current research. Consequently, the development of more efficient pathways for mineralization technology has emerged as a critical challenge within scientific research. I propose that integrating various advanced technologies could yield innovative strategies for the comprehensive degradation of organic pollutants, potentially overcoming current technical bottlenecks and fulfilling the dual objectives of environmental protection and water quality enhancement.

## 5. Concluding Remarks and Future Perspectives

This paper conducts a systematic analysis of the processes and efficiencies associated with the US/PI system for degrading organic pollutants in water. Initially, the paper delineates the intrinsic degradation mechanisms of the US/PI system and explains the underlying theory of this technology in a clear and accessible manner. Subsequently, the paper examines how critical operating parameters, such as ultrasonic power, pH, reaction temperature, PI concentration, inorganic ions, and dissolved organic matter in wastewater, influence the removal efficiency of the US/PI system. Finally, the paper summarizes the high-efficiency degradation of organic pollutants in wastewater using the US/PI system in practical applications. Overall, the US/PI composite system undoubtedly demonstrates significant potential and is poised to become a leading technical solution for treating organic pollutants in diverse wastewater types. However, research on pollutant degradation in wastewater treatment using the US/PI system remains nascent. To further advance scientific research and technological innovation in this area, the author identifies critical needs and directions for future research:

(a) It is advisable to employ innovative experimental designs and precise instrumentation, such as experimental segmentation and variable control, to differentiate and accurately quantify the contributions of thermal decomposition processes and free radical reactions. Concurrently, there is an urgent need to develop new technological approaches for the direct identification of various free radicals within the US/PI system, such as employing electron spin resonance (ESR) spectroscopy. Furthermore, these advancements will enable a comprehensive elucidation of the mechanisms by which free radicals degrade organic pollutants.
(b) Although mineralization levels can initially be gauged using Total Organic Carbon (TOC) and Chemical Oxygen Demand (COD), the toxicity of intermediates produced during degradation processes holds significant importance. The discernible scarcity of research focusing on biotoxicity and bioindicators like Biochemical Oxygen Demand (BOD) heralds significant opportunities for future investigations.
(c) The degradation of organic pollutants via the US/PI system involves an inherently intricate and multifaceted mechanism. While this manuscript has delved into aspects of free radical oxidation and ultrasonic pyrolysis, numerous unexplored facets remain, necessitating systematic investigation through further experimental research.
(d) While the US/PI system has demonstrated superior efficacy in the degradation of organic pollutants relative to traditional methodologies, its elevated economic cost remains a significant impediment to widespread adoption. Consequently, identifying strategies to mitigate costs while preserving the system’s high efficiency constitutes a crucial research trajectory for the advancement of US/PI technologies.
(e) Currently, the application of the US/PI system is constrained by operating conditions, including ultrasound frequency and intensity, as well as PI concentration. Future research should explore optimizing these conditions to maximize degradation efficiency, while also considering the balance between cost and energy consumption.
(f) The majority of research on the degradation of organic pollutants in wastewater using the US/PI system remains at the laboratory stage. Future research should investigate scaling up the US/PI system for industrial applications, including treating complex combinations of organic pollutants in real-world wastewater scenarios and assessing the system’s stability and sustainability.
(g) When employing the US/PI system for wastewater treatment, it is crucial to evaluate the by-products generated during the reaction and their potential environmental impacts. Future research should encompass the identification, quantification, and toxicological evaluation of these by-products.
(h) Future research should investigate integrating the US/PI system with other wastewater treatment methodologies, such as biological treatment and adsorption, to enhance treatment efficacy and cost-efficiency. This integrated approach could potentially be more effective in treating recalcitrant or highly concentrated organic pollutants.

## Figures and Tables

**Table 1:** Comparison of advantages and disadvantages of mainstream advanced oxidation technologies.

Technologies	Advantages	Disadvantages	References
**US/PI oxidation method**	Efficient degradation of pollutants; rapid reaction kinetics; outstanding environmental compatibility; wide applicability; straightforward operation.	Substantial energy consumption; significant equipment expenditure.	[22]
**Photochemical catalytic oxidation method**	Lighting is sustainable and eco-friendly; exerts minimal impact on water quality; characterized by low operating costs; boasts a long service life	Optimization is required for its integration with other advanced technologies such as UV/H_2_O_2_ and UV/PDS; the UV radiation from built-in light tubes poses health risks to humans.	[23]
**Electrochemical oxidation process**	The electrolysis device is streamlined, user-friendly, and offers straightforward control, with an anode that efficiently oxidizes pollutants. Altering the anode material enables the targeted degradation of various organic compounds.	The energy consumption is excessively high, the reactor exhibits suboptimal efficiency, and the associated equipment costs are considerable.	[24]
Ozone technology	Fast oxidation speed and high oxidation efficiency.	The equipment configuration is intricate and characterized by high energy demands accompanied by significant financial outlays.	[9]

**Table 2:** Degradation mechanisms of different organics in the US/PI system.

Contaminant	Degradation Products	Key Mechanism	End Products	References
** Perfluorooctanoic acid **	Perfluoroolefins. 1 h-perfluoroalkanes	Cleavage of C–C and C–F bonds in perfluoroanions; ultrasonic pyrolysis occurring at or near the bubble interface; oxidation of PFOA via electron transfer mediated by IO_3_^•^ radicals.	CO, CO_2_, HF, etc.	[34]
** Acid Blue 92 **	1,2-Benzenedicarboxylic acid 1-Hydroxycyclohexane-1-carboxylic acid Aniline; acetic acid; formamide	Dyes undergo degradation through the cleavage of N≡N, C-C, C-S, and C-N bonds. Hydroxyl and amino groups attached to the benzene ring undergo oxidation. The aromatic ring undergoes opening, leading to the formation of the final product.	CO_2_, H_2_O, etc.	[35]
** landfill leachate **	Cyclohexylsiloxane Cyclotridecane Phenol	IO_3_^•^, IO_4_^•^ and • OH radicals oxidize litter permeability, forming intermediates that are finally broken down into CO_2_ and H_2_O.	CO_2_, H_2_O, etc.	[36]
** acid orange 7 **	1,3-Biphenyl Acetic acid 2-propionic acid Methyl ethyl Cyclohexene Ethylbenzene Trimethyl	Cleavage of the C–N bond in heterocyclic compounds; extraction of hydrogen atoms. oxidation of methyl groups; nitro group hydrolysis on aromatic rings; oxidation of hydroxyl groups and phenolic compounds; internal ultrasound-assisted pyrolysis.	NH_3_, SO_2_ CO_2_, H_2_O, etc.	[37]
industrial wastewater (TAAs)	Trimethylaniline, aniline, o-toluidine, o-aminoaniline, xylene, ethylbenzene, and duran	Cleavage of azo groups, as well as methyl, ethyl, and C-H-O bonds; ultrasound-assisted internal pyrolysis; oxidation of hydroxyl and amino groups on benzene rings; oxidation and decarboxylation of methyl groups, accompanied by hydrogen extraction; hydrolysis of aromatic rings.	NH_3_, CO_2_, etc.	[38]

**Table 3:** The impact of pH levels on the degradation of organic matter within the US/PI framework.

Analytes	Selected pH Range	Optimal pH	Proposal Reactions	References
Perfluorooctanoic acid	3.9, 7, 10.1	3.9	$2H^{+}IO_{4}^{-}\overset{)))}{\to }IO_{3}^{•}+H_{2}O$	[34]
Brilliant Blue R	2, 5.2, 8	8	$IO_{3}^{•}+IO_{4}^{-}\overset{)))}{\to }IO_{3}^{-}+IO_{4}^{•}$ $O_{2}+IO_{4}^{-}+OH^{-}\overset{)))}{\to }IO_{3}^{-}+H_{2}O+2O_{2}^{•-}$ 2H2O+2O2•−→)))2OH−+H2O2+O21	[40]
Bisphenol A	6, 7, 8.5	8.5	$IO_{3}^{•}+IO_{4}^{-}\overset{)))}{\to }IO_{3}^{-}+IO_{4}^{•}$ $O_{2}+IO_{4}^{-}+OH^{-}\overset{)))}{\to }IO_{3}^{-}+H_{2}O+2O_{2}^{•-}$ 2H2O+2O2•−→)))2OH−+H2O2+O21	[24]
Phenol	3, 7, 11	3	$•H+IO_{4}^{-}\overset{)))}{\to }IO_{3}^{-}+•\mathrm{OH}$ $•H+IO_{3}^{-}\overset{)))}{\to }IO_{2}^{-}+•\mathrm{OH}$ $•H+IO_{4}^{-}\overset{)))}{\to }IO_{3}^{•}+OH^{-}$	[46]
Garbage permeate	3, 5, 7, 9, 11	3	$IO_{4}^{-}\overset{)))}{\to }IO_{3}^{•}+O^{-•}$ $O^{-•}+H^{+}\overset{)))}{\to }OH^{•}$ $OH^{•}+IO_{4}^{-}\overset{)))}{\to }IO_{4}^{•}+OH^{-}$ $OH^{•}+IO_{3}^{-}\overset{)))}{\to }\mathrm{HI}O_{4}^{-}$ $2OH^{•}\overset{)))}{\to }H_{2}O_{2}$ $2IO_{4}^{•}\overset{)))}{\to }I_{2}O_{8}$ $I_{2}O_{8}+H_{2}O_{2}\overset{)))}{\to }IO_{3}^{-}+IO_{4}^{-}+2H^{+}+O_{2}$ $2IO_{3}^{•}\to \overset{)))}{\to }I_{2}O_{6}$ $I_{2}O_{6}+H_{2}O\to \overset{)))}{\to }IO_{3}^{-}+IO_{4}^{-}+2H^{+}$ $O_{2}+O\overset{)))}{\to }O_{3}$ $O_{3}+IO_{3}^{•}\overset{)))}{\to }IO_{4}^{•}+O_{2}$	[36]

**Table 4:** A comprehensive overview of removal efficiencies for various organic pollutants via the US/PI system.

Contaminant	Ultrasound Properties	PI Concentration	US Removal Efficiency (%)	PI Removal Efficiency (%)	US/PI Removal/TOC Efficiency (%)	References
Perfluorooctanoic acid (170.1 μmol/L)	P:120 W f:40 kHz	45 mmol/L	2 h/26.2	2 h/2	2 h/96.5/95.7	[34]
Brilliant Blue R (5 mg/L)	P:80 W f:300 kHz	10 mmol/L	140 min/100	60 min/0	60 min/100/100	[40]
Acid Blue 92 Dye (10 mg/L)	P:150 W f:36 kHz	0.15 mol/L	150 min/46	none	150 min/98/none	[35]
Tetracycline (50 mg/L)	P:256 W f:37 kHz	0.01 mol/L	45 min/12.8	none	45 min/94.2/77.9	[66]
Orange 7 Acid (50 mg/L)	P:150 W/L f:36 kHz	2 mmol/L	105 min/27.5	60 min/0	90 min/96/none	[37]
Industrial wastewater (2880 mg/L)	P:100 W f:37 kHz	0.11 mol/L	120 min/18.43	none	120 min/98/79	[45]
Ramazol Black B (100 mg/L)	P:500 W f:60 kHz	10 mg/L	100 min/34.7	none	100 min/98.7/none	[48]
Aromatic amine (1990 mg/L)	P:500 W f:35 kHz	16 mg/L	150 min/79	none	150 min/99/96	[38]

## Data Availability

The data are contained within the article.
